# Gestational Paracetamol (Acetaminophen) Toxicity Induces Behavioral and Structural Brain Defects in Rats

**DOI:** 10.3390/cimb48070714

**Published:** 2026-07-13

**Authors:** Linah H. Ali, Hanaa A. Khalaf, Sameera S. Hamed, Ahmad M. Helaly, Aya E. Maghrabia, Doaa Ghorab, Amal A. El Bakary

**Affiliations:** 1 Forensic Medicine and Clinical Toxicology Department, Faculty of Medicine, Mansoura University, Mansoura 35516, Egypt; 2 Medical Histology and Cell Biology Department, Faculty of Medicine, Mansoura University, Mansoura 35516, Egypt; 3 Department of Histology, Hours University, New Damietta City 8027101, Egypt; 4 MERC, Faculty of Medicine, Mansoura University, Mansoura 35516, Egypt; 5 Pathology Department, Faculty of Medicine, Mansoura University, Mansoura 35516, Egypt

**Keywords:** paracetamol, prenatal exposure, BDNF, immunohistochemistry, neurodevelopmental disorders, autism

## Abstract

Paracetamol is a well-known analgesic drug. Studies linked gestational paracetamol use to disorders like autism and attention-deficit/hyperactivity disorder. This study aimed to evaluate the role of prenatal paracetamol exposure in 1-month-old offspring rats. Sixteen pregnant albino rats were divided into four groups: group I received saline; group II received sodium valproate 600 mg/kg i.p. on embryonic day 13; group III received paracetamol 100 mg/kg i.p. daily from embryonic day 13 to 21; group IV received paracetamol 300 mg/kg i.p. on embryonic day 13. Offspring were assessed for behavioral test parameters. Animal brains underwent histopathological and immunohistochemical analysis for brain-derived neurotrophic factor (BDNF). Offspring in groups II (valproic acid) and IV (single paracetamol dose) showed differences in neurobehavioral test parameters, supported by cerebellar and hippocampal pathology. BDNF-stained cerebellum and hippocampus sections from the valproic group and the single paracetamol group revealed focal staining in granular cerebellar cells and pyramidal hippocampal cells. These findings indicate that prenatal exposure to high doses of paracetamol induces neurodevelopmental pathology. The safety of paracetamol use during pregnancy requires further evaluation.

## 1. Introduction

Paracetamol, also known as N-acetyl-p-aminophenol (APAP), is a widely used analgesic and antipyretic drug [1]. It possesses analgesic and antipyretic properties with minimal peripheral anti-inflammatory activity. It is mainly prescribed for the treatment of mild to moderate pain or fever [2].

Studies evaluating therapeutic doses of paracetamol have generally failed to demonstrate adverse fetal effects during any trimester of pregnancy. Paracetamol has traditionally been classified as a category B medication (drugs with no risk in humans), as many authors stated that paracetamol, as a single agent, does not increase fetal risk in any trimester and is considered safe for use in pregnancy [3,4,5,6,7]. However, increasing evidence has raised concerns regarding paracetamol use during pregnancy, as the drug crosses the placenta and may affect the developing fetus. Although relatively few studies have investigated paracetamol misuse, growing evidence suggests that chronic and maternal abuse of paracetamol is associated with hypertension, kidney, and lung disorders [8,9]. An important epidemiological study demonstrated that excessive maternal exposure to paracetamol was associated with behavioral changes in children, including reduced shyness [10]. Another study recorded the association between excessive maternal exposure to paracetamol and the development of attention-deficit/hyperactivity disorder in siblings [11].

During pregnancy, paracetamol clearance increases significantly. Thus, the analgesic effect of paracetamol will decrease more rapidly, potentially necessitating higher doses to achieve adequate analgesic effects [2]. Paracetamol toxicity can result from either an acute overdose or chronic overuse [12].

In 2015, the Food and Drug Administration (FDA) issued a report regarding the use of pain medications during pregnancy. The report revealed that the evidence regarding the possible connection between paracetamol and attention-deficit hyperactivity disorder was inconclusive [13].

Paracetamol can cross the blood–brain barrier because of its low molecular weight and low affinity for plasma proteins [14].

Neurobehavioral disorders encompass a wide range of impairments associated with other conditions that cause brain disease or injury. These disorders are characterized by interruptions or disturbances in the acquisition of skills across a variety of developmental domains, including motor, social, language, and cognitive aspects. Neurobehavioral disorders may manifest as attention disorders, autistic spectrum disorders, and motor and sensory processing disorders [15].

Over the past decade, there has been a rapid increase in the incidence of childhood neurodevelopmental disorders, like attention-deficit/hyperactivity disorder (ADHD). It has been suggested that this increased rate is not attributable solely to changes in diagnostic procedures or parental awareness, but also to non-genetic risk factors acting during the prenatal period [16]. The increasing prevalence of these disorders may partly result from prenatal exposure to infections or certain drugs, such as rubella, or, like valproic acid, alcohol, or cocaine during pregnancy [17].

BDNF (brain-derived neurotrophic factor) plays multiple roles in neuroprotection and is a good indicator of neurogenesis. BDNF has been implicated in various neurological and neurodevelopmental disorders. In animals, locomotor and behavioral responses may be altered by therapeutic doses of paracetamol administered during early development. These alterations may be mediated through paracetamol-induced changes in BDNF and neurotransmitter systems (including serotonergic, dopaminergic, and adrenergic systems, as well as the endogenous endocannabinoid system), and cyclooxygenase-2 [18].

This study aimed to evaluate the effects of prenatal exposure to paracetamol at therapeutic and high single doses on the brains of 1-month-old rat offspring. The study combined behavioral test parameters, histopathology, and immunohistochemistry to assess evidence of paracetamol-induced autism-like behavioral changes in animals. The positive control group consisted of offspring prenatally exposed to valproic acid, a well-established experimental model of autism [19]. The present study also evaluated BDNF immunostaining in cerebellar and hippocampal tissue.

## 2. Materials and Methods

This experimental study was approved by the Medical Research Ethics Committee, Faculty of Medicine, Mansoura University, Mansoura City, Egypt, by code number (MS/May 2017).

### 2.1. Materials

#### 2.1.1. Chemicals

1. Paracetamol: In the form of an **Injectmol** infusion bottle (100 mL) in a concentration of 10 mg/mL, was purchased from Pharco B International Egypt Company (Alexandria, Egypt).
2. Sodium valproate: In the form of Depakine ampoule (4 mL) in a concentration of 100 mg/1 mL, was purchased from Sanofi Aventis France Company(Paris, France).
3. Brain-derived neurotrophic factor antibody (BDNF) (N-20): SC-546 is a purified rabbit polyclonal IgG raised against a peptide mapping to an internal region of human BDNF, as used in rats. It was purchased from Santa Cruz Biotechnology(Dallas, TX, USA). BDNF is a cellular signal that is looked at as a diffuse reaction dispersed through the nerve cell cytoplasm and does not appear in the nucleus.

#### 2.1.2. Animals

This study was conducted on 16 pregnant albino Wistar rats during early gestation. The rats were obtained from the animal house of the Medical Experimental Research Center (MERC) at the Faculty of Medicine, Mansoura University. They weighed about 150–200 g. Animals were kept in sanitary cages under normal laboratory conditions, including a 12 h light/dark cycle and good aeration. Room temperature was maintained at 18–22 °C. The rats were fed standard laboratory food and tap water. The animals were sacrificed by a single overdose of thiopental sodium (7.5 mg/100 g body weight) administered intraperitoneally. The offspring were allowed to grow for one month and were then sacrificed using the same protocol. Both sexes were selected in the study. Positive autistic findings in females suggest an aggressive toxic model of the disease.

### 2.2. Experimental Design

The experimental work was conducted at the Medical Experimental Research Center (MERC), Faculty of Medicine, Mansoura University, Mansoura City, Egypt.

Pregnant rats were divided into four groups; each group contained four rats:

**Group I (negative control group):** They received an i.p. injection of saline once daily for 9 days from embryonic day 13 to 21.

**Group II (positive control group):** They received sodium valproate at 600 mg/kg once i.p. at embryonic day 13.

**Group III:** They were given a dose of paracetamol as 100 mg/kg i.p. daily for 9 days from embryonic day 13 to 21 (late 2nd trimester and 3rd trimester in which brain development occurs).

**Group IV:** They were given a single high dose of paracetamol (300 mg/kg i.p.) at embryonic day 13.

### 2.3. Methods

After 1 month of delivery, all offspring were examined by a trained observer for various behavioral parameters using the open-field test in an ANY-maze. Then, the offspring were sacrificed; the brains were removed and examined histopathologically and immunohistochemically.

#### 2.3.1. Neurobehavior Test Parameters

The ANY-box^®^ (Stoelting Company, Wood Dale, IL, USA) was a multi-configuration behavioral apparatus designed to automate a range of standard neurobehavioral test parameters. It consisted of two components: an ANY-box base and core. The ANY-box base was attached to a camera to track the animals. The ANY-box core consisted of the ANY-maze software (an elastic video-tracking system designed to computerize tests in locomotor experiments) and an ANY-maze interface. The open field test was the neurobehavioral test parameter used in this study, as it is widely used to assess locomotor activity in offspring across all experimental rat groups at one month of age. This examination was carried out in a completely isolated environment, free of external noise. Each animal was placed individually at the center of the apparatus and observed for five minutes. The apparatus floor was cleaned with 70% ethyl alcohol between each rat. Each rat trial was recorded by a camera fixed above the apparatus. The open field test measured neurobehavioral function for each animal by the following parameters:

1. Total distance traveled (in centimeters).
2. Average speed (centimeters per second).
3. Total time being mobile (in seconds).
4. Number of the mid-zone crossing (line crossing).
5. Immobile, freezing, and mobile episodes.
6. Clockwise and anticlockwise rotations.

#### 2.3.2. Histopathological Evaluation

After the neurobehavioral test parameters were done and under deep anesthesia, the offspring were sacrificed. The brain was dissected, processed, and stained with hematoxylin and eosin. Hippocampus and cerebellum were examined microscopically and photographed.

#### 2.3.3. Immunohistochemical Study

Paraffin-embedded sections were immunohistochemically stained with BDNF antibody at a dilution of 1/100. According to the manufacturer, sections were mounted on positively charged slides and then immunoassayed by the avidin-biotin technique. The sections were deparaffinized, rehydrated, and then washed with tap water. Endogenous peroxidase activity was blocked by incubating the section in 0.01% H_2_O_2_, and the antigen retrieval was performed by incubating the section in 0.01 M citrate buffer (pH 6) for 30 min. To control nonspecific background, the sections were incubated for 20 min in diluted normal rabbit serum, then incubated in diluted primary antibody (BDNF, rabbit polyclonal antibody) at 1/100 for 2 h. After that, the slides were incubated with the avidin-biotin complex (ABC) substrate for 1 h, followed by incubation in peroxidase substrate solution for 6–10 min. Finally, the sections were counterstained with hematoxylin. The primary antibody was replaced with phosphate-buffered saline for a negative control slide.

#### 2.3.4. Digital Morphometric Study

Slides were photographed using an Olympus^®^ digital camera (E24-10-megapixel, Beijing, China) mounted on an Olympus^®^ microscope (Olympus^®^ model CX31RTSF, Tokyo, Japan) with a 1/2 X photo adaptor, via a 40× objective lens. The obtained photos were examined on an Intel^®^ Core I3^®^-based computer using Video Test Morphology^®^ software (Saint Petersburg, Russia) with a built-in routine specifically designed to estimate the percentage of BDNF-positive expression in the area. Using ImageJ-Win64 software, the number of nuclei in the pyramidal layer of the CA1 region of the hippocampus and their average size are measured by converting the image to 8-bit, adjusting brightness/contrast, setting a threshold, processing the binary image, applying watershed segmentation, and analyzing particles. Also, three measurements of pyramidal cell layer thickness in the CA1 region of the hippocampus were taken using the straight-line tool, and the average for each group was calculated.

### 2.4. Statistical Analysis

Statistics were analyzed via the computerized statistical package for the social sciences (SPSS) version 16. Normally distributed quantitative data were described by the mean and standard deviation. Quantitative data that were not normally distributed were described using median and range (minimum-maximum). A *p*-value ≤ 0.05 was considered statistically significant, and a highly significant difference at *p*-value < 0.001. Therefore, the following tests were used:

1. Kruskal–Wallis test: to compare quantitative data that was not normally distributed between more than two groups.

2. The Mann–Whitney U test was used to compare quantitative data that are not normally distributed between two groups.

One-way ANOVA was used to test for differences in normally distributed quantitative data across three or more groups, followed by Bonferroni multiple comparisons.

## 3. Results

### 3.1. Neurobehavior Test Parameters

Figure 1 shows that the offspring of group II and IV had significantly lower mobility episodes when compared with the offspring of group I (p1 = 0.009, p3 = 0.05, respectively) and had no significant difference when compared with the offspring of group III (p4 = 0.07, p6 = 0.2). However, the offspring of Groups II and IV exhibited significantly higher immobility (p1 = 0.001, p3 = 0.003) and freezing episodes (p1 = 0.01, p3 = 0.01) than those of Group I. Similarly, the offspring of Groups II and IV showed significantly higher immobility (p4 = 0.001, p6 = 0.006) and freezing episodes (p4 = 0.03, p6 = 0.02) than those of Group III.

No statistically significant difference was detected between the offspring of group II and of group IV (p5 = 0.2, 0.09, 0.9, 0.8) regarding these neurobehavioral test parameters (mobile, immobile, and freezing episodes as well as total mobility time, respectively). Also, no statistically significant difference was detected between the offspring of groups I and III (p2 = 0.9, 0.9, 0.9, 0.2) regarding these neurobehavioral test parameters (mobile, immobile, and freezing episodes, as well as total mobility time, respectively).

Figure 2 shows significantly lower distance traveled (p1 = 0.03, p3 = 0.02), average speeds (p1 = 0.001, p3 = 0.001), line crossing (p1 < 0.001), and clockwise rotations (p1 < 0.001, p3 = 0.005) among offspring of group II and IV, respectively, when compared with those of group I. Also, significantly higher distance traveled (p4 = 0.03, p6 = 0.02), average speeds (p4 = 0.007, p6 = 0.01), line crossing (p4 = 0.001, p6 = 0.002), and clockwise rotations (p4 = 0.001, p6 = 0.002) were detected among offspring of group II and IV, respectively, compared to group III. No statistically significant difference was detected between the offspring of group II and of group IV (p5 = 0.9, 0.5, 0.6, 0.07, 0.1) regarding the neurobehavioral test parameters (distance traveled, average speed, line crossing, clockwise rotation, and anticlockwise rotation of the animal body, respectively). Also, no statistically significant difference was detected between the offspring of group I and of group III (p2 = 0.9, 0.6, 0.9, 0.3, 0.2) regarding the neurobehavioral test parameters (distance traveled, average speed, line crossing, clockwise rotation, and anticlockwise rotation of the animal body, respectively.

Figure 3 discusses the degree of BDNF staining of hippocampus regions in the offspring. Both the Valproic acid positive control group and the single-dose paracetamol group expressed positive staining. The repeated low-dose paracetamol group showed no significant increase in BDNF staining.

### 3.2. Histopathological Evaluation

#### 3.2.1. Hippocampus

The normal histological architecture of the hippocampus of group I (negative control group) offspring was observed by light microscopy of H&E-stained sections. The hippocampus is composed of the proper hippocampus, the dentate gyrus, and the subiculum. The hippocampus consists of the cornu ammonia (CA), which comprises CA1, CA2, CA3, and CA4, and continues as the subiculum. CA4 was surrounded by a dark C-shaped dentate gyrus (Figure 4). CA1 was composed of molecular, pyramidal, and polymorphic regions. The pyramidal layer included 4–5 dense layers of small pyramidal cells with vesicular nuclei. The molecular and polymorphic layers were relatively cell-free and contained blood vessels (Figure 5A). The dentate gyrus comprises molecular, granular, and polymorphic areas. The granular area formed the principal layer. It had extreme cell density, formed by compact columns of rounded granule cells with vesicular nuclei, and contained blood vessels. The polymorphic layer contained some pyramidal cells and blood vessels (Figure 5B).

Examination of H&E-stained sections of the hippocampus of group II (valproic acid-treated group) offspring showed distinct histological changes seen only at the pyramidal layer in the CA1 region, which appeared attenuated and irregular with focal cell loss (Figure 5C). Meanwhile, the dentate gyrus appeared normal with normal thickness of all its layers (Figure 5D).

Examination of H&E-stained sections of the hippocampus of group III (therapeutic dose of paracetamol-treated group) offspring showed mostly normal hippocampi, with the same general picture of CA1 (Figure 5E) and the dentate gyrus (Figure 5F) as detected in the offspring of group 1 (negative control group).

Examination of H&E-stained sections of the hippocampus from group IV (toxic dose of paracetamol-treated) offspring showed focal mild attenuation of the pyramidal cell layer in the CA1 region (Figure 5G). At the same time, the dentate gyrus appeared normal with normal thickness of all its layers (Figure 5H).

Using the image analysis application (ImageJ-win64) revealed that the highest number of nuclei in the pyramidal layer of the hippocampal CA1 region was in groups I and III, while it showed decreased numbers in groups II and IV, with nearly the same count (Table 1). Also, the highest average pyramidal layer of hippocampal CA1 region thickness was found in groups I and III, while the thickness of groups II and IV was much reduced (Table 2).

#### 3.2.2. Cerebellum

A study of H&E-stained sections of the cerebellum of group I (negative control group) offspring showed the usual cerebellar structural design. The cerebellar cortex consists of three distinct layers: the outer molecular layer, the middle Purkinje layer, and the inner granular layer. The granular cell layer was densely packed with cells; it differs from the molecular layer, which is considered cell-free. The Purkinje layer was composed of Purkinje cells arranged in a single row between the molecular and granular layers. Those cells had large, pyriform-shaped cell bodies, centrally located vesicular nuclei, and apical dendrites extending upward into the molecular layer. The granular layer was composed of numerous small, closely packed granular cells having dark spherical nuclei (Figure 6A).

A study of H&E-stained sections of the cerebellum of group II (valproic acid-treated group) offspring showed a decreased number of the Purkinje cells due to cell loss (crossed arrows) and its shrinkage, which became smaller and pyknotic (arrows) with normal vasculature (curved arrow) at the molecular layer but showed mild capillary congestion at the white matter (WM) layer (Figure 6B).

The microscopic examination of specimens of the offspring of group III (therapeutic dose of paracetamol-treated group) revealed mostly normal cerebellar architecture, as the same general picture of the cerebellum of the offspring of group 1 (negative control group) (Figure 6C).

Microscopic examination of specimens of the offspring of group IV (toxic dose of paracetamol-treated group) showed a decreased number of the Purkinje cells due to cell loss and their shrinkage, which became smaller and pyknotic as seen in group II (valproic acid-treated group) (Figure 6D).

The approximate number of Purkinje cells in the cerebellum was 11 cells in Group I, three cells in Group II, 10 cells in Group III, and four cells in Group IV. Purkinje cell counts were determined manually from representative photomicrographs obtained from each group.

### 3.3. Immunohistochemistry Evaluation

Normally, the BDNF is strongly expressed at the dentate gyrus and weakly expressed focally at the cornu ammonia, so the photos are taken at CA3. So, immunostained sections of the hippocampus of the offspring of group I (negative control group) and group III (therapeutic dose of paracetamol-treated group) revealed a weak focal reaction of BDNF in the cytoplasm of the granular cells and around blood vessels (Figure 7a and Figure 7c, respectively).

Immunostained sections of the hippocampus of the offspring of group II (valproic acid-treated group) and group IV (toxic dose of paracetamol-treated group) revealed focal increases in BDNF immunoreactivity in the cytoplasm of granular cells and around blood vessels (Figure 8b and Figure 8d, respectively). Immunostained sections of the cerebellum of the offspring of group I (negative control group) and III (therapeutic dose of paracetamol-treated group) revealed no reaction of BDNF in the cytoplasm of the Purkinje cells or around blood vessels (Figure 8a and Figure 8c, respectively). Immunostained sections of the cerebellum of offspring of group II (valproic acid-treated group) and group IV (toxic dose of paracetamol-treated group) revealed no BDNF reactions in the cytoplasm of the Purkinje cells in comparison with focal positivity for granular cells and blood vessels pointed out by black arrows (Figure 8b and Figure 8d, respectively).

Statistical comparison between percentage areas of BDNF immunohistochemically stained among the examined groups showing the percentage (%) area of BDNF immunohistochemically stained is significantly higher among offspring of groups II and IV when compared with offspring of group I (p1 ≤ 0.001, p3 ≤ 0.001, respectively) or with that of group III (p4 ≤ 0.001, p6 ≤ 0.001). However, the % area of BDNF immunoreaction shows no statistical difference among the offspring of group IV when compared with that of group II (p5 = 0.9) and no statistical difference between the % offspring of group III and that of group I (p2 = 0.2) (Figure 6).

## 4. Discussion

The present study was done to assess prenatal exposure to paracetamol by two chronic and high single doses, aiming to model gestational abuse and toxicity of paracetamol. In the present study, the neurobehavioral test parameters in the offspring of groups II and IV showed significantly lower distance traveled, average speed, mobile episodes, line crossing, and clockwise rotations than those in the offspring of groups I and III. Similarly, immobile and freezing episodes were significantly higher among the offspring of groups II and IV when compared with the offspring of groups I and III. Also, no significant differences were observed between the offspring of groups II and IV, nor between the offspring of groups I and III, according to these tests.

The histopathological examination showed a relation with the behavior test parameters in both the valproic group and the high-dose paracetamol group, showing reductions in pyramidal and granular cells in hippocampal samples. Similar adverse responses were observed in cerebellar samples involving the Purkinje cell population. These pathological findings indicated cell injury and apoptotic responses due to the toxicity model. It is suggested that the autistic-like behavioral responses observed in the animals may be related to neuronal loss and functional impairment. BDNF immunohistochemistry showed focal positive cells in the granular cells of the hippocampus in the valproic autistic model and, to a lesser extent, in the high-dose paracetamol group. The present study demonstrated Purkinje cell loss and degenerative changes, and possible excitation of the granular cell population despite this apoptosis. These findings were related to the focal expression of the BDNF in the granular cells. It was suggested that autism pathogenesis was correlated with increased cerebellar excitability [20,21]. Increased BDNF expression and pyramidal cell activity may contribute to behavioral alterations associated with hyperactivity and ADHD-like features. It was reported that low chronic paracetamol exposure improved water maze tests (neurobehavior test parameter), while high doses caused spatial memory deficits in the water maze. Also, proposed that the usage of paracetamol during pregnancy corresponded to neurodevelopmental disorders like hyperkinetic disorder, attention-deficit/hyperactivity disorder (ADHD) [22,23].

The high single dose of paracetamol demonstrated valproic-like effects, suggesting a similar mechanism. The present study proposed the direct effects of valproic acid and paracetamol on animal brains. A similar concept was adopted by other work, as the paracetamol toxic hepatic dose killed the animal by hepatic failure before affecting the brain. So, the released free radicals are unlikely to be the cause of the current model cell injury [24]. The present work suggests that high single doses of both valproic and paracetamol induced a direct process that could contribute to the development of neurodevelopmental disorders like autism and hyperactivity [25,26]. The focal expression of BDNF may suggest neurogenesis and possible stem cell activation in response to the observed pathology [27]. Meta-analysis studies demonstrated a high serum level of BDNF in autism, unlike schizophrenia, and it has been suggested that BDNF is not merely a response marker but may also be involved in the pathogenesis of the disorder [28,29].

Experimental work examined long-term prenatal exposure (for more than 28 days) to therapeutic doses of paracetamol on neurodevelopmental outcomes in children at 3 months of age. They found that these children had poorer gross motor development and communication skills and also exhibited more hyperactivity [30]. Moreover, it was reported that maternal therapeutic dose of paracetamol during pregnancy was linked with an increased risk of hyperkinetic disorders and ADHD-like behaviors, and autism [31,32].

The hippocampus is known to be very important for cognitive development in children, including language syntax, semantics, and pragmatics, as well as creativity and behavior. Autism was suggested to be a developing disorder of hippocampal dysfunction [33]. Although the model used in the study does not fully align with routine paracetamol use in clinical practice, the results strongly correlated with the pathology observed in the animals. These findings in combination raise questions about the use of paracetamol in pregnancy. It is suspected that paracetamol exposure, particularly when combined with other environmental or inflammatory factors, may increase the risk of neurodevelopmental abnormalities. A new study used BTBR mice to evaluate autistic characteristics using the Novel Object Recognition Test (NORT) and the Open Field Locomotor Test (OFT). The NORT assesses memory function in the animal model, whereas the OFT measures anxiety and hyperactivity [34]. An important rat study raised concerns about autism, as maternal exposure to acetaminophen correlated with the pathology of the cannabinoid receptors. This study supports the findings of the present research. Further multi-omic studies to correlate BDNF with other receptors like cannabinoids or serotonin ones are needed [35].

Increasing evidence suggests an association between acetaminophen exposure and neurodevelopmental disorders, and such assumptions depend on statistical evidence derived from clinical and animal modeling that supports the potential role of acetaminophen in the pathogenesis of autism [36]. One limitation of the study is that we did not focus on cortical pathology; however, a previous study showed similar cell injury and apoptosis in the cortex as a direct effect of paracetamol, in a dose-dependent manner [37].

## 5. Conclusions

According to the results of the present work, it can be concluded that prenatal exposure to paracetamol in high doses can induce disturbances in the neurobehavioral test parameters and histopathological changes in the hippocampus and the cerebellum in rats, which resemble certain behavioral and neuropathological features associated with autism spectrum disorders and hyperactivity. The present study demonstrated increased BDNF expression in the hippocampal sections of experimental animals. The observed effects of paracetamol were comparable to those induced by valproic acid, the well-known autism model in animals. The mechanisms underlying these adverse effects require further investigation.

## Figures and Tables

**Figure 1:**
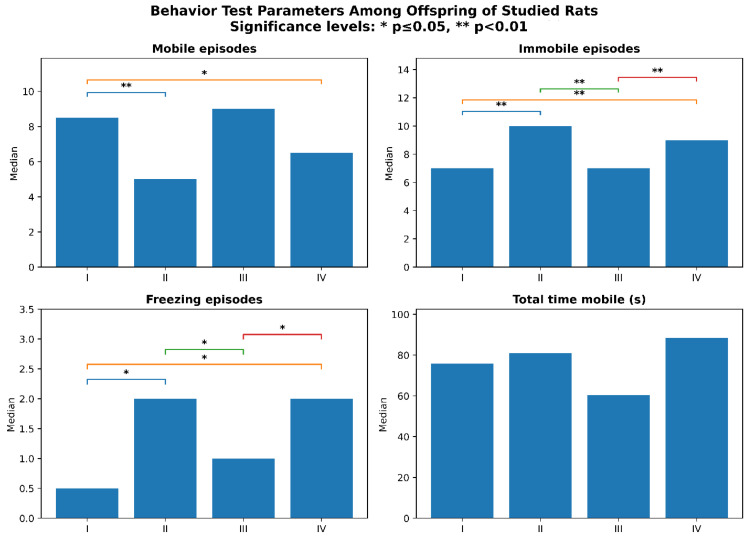
Summarizes the results, where the categories are the 4 groups of the study in comparison to mobile, immobile, freezing episode, and total time mobile, respectively. Group 1 has 8 animals, group 2 has 7 animals, group 3 has 7 animals, and group 4 has 8 animals. * indicates significance.

**Figure 2:**
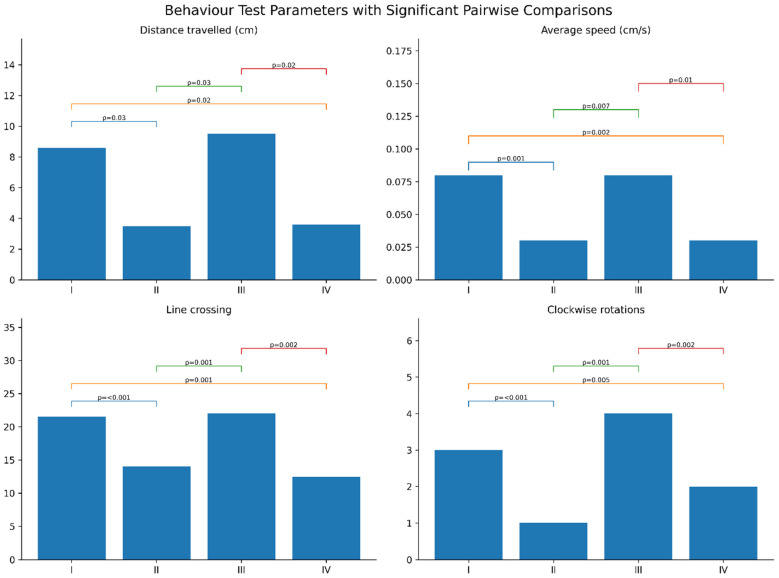
Summarizes the behavioral test parameters (distance traveled, average speeds, line crossing, and rotations) seen in among the different groups; min: minimum, max: maximum, n: number, s: seconds, *p*-values indicate comparisons between groups assessed via Kruskal–Wallis test, p1 value: on comparing sibling of group I and group II, p2 value: on matching sibling of group I and group III, p3 value: on comparing sibling of group I and group IV, p4 value: on comparing sibling of group II and group III, p5 value: on comparing sibling of group II and group IV, p6 value: on comparing sibling of group III and group IV.

**Figure 3:**
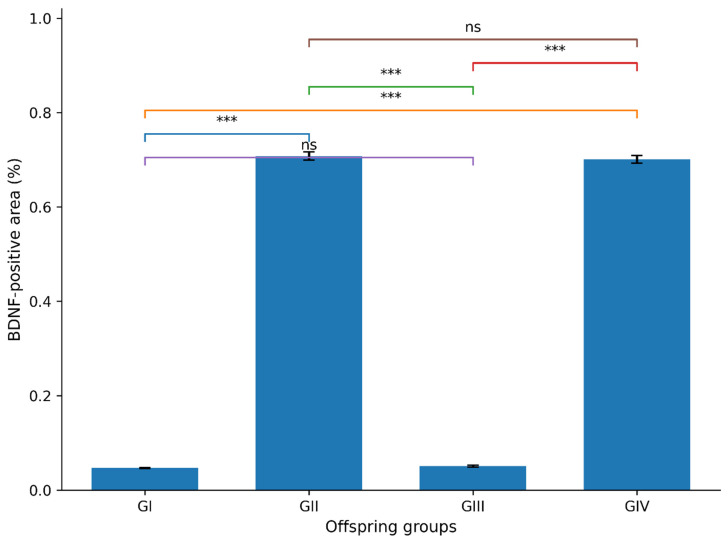
Explains the data, where groups II (valproic) and IV (high paracetamol) showed positive BDNF staining in comparison to the control groups. This figure indicates that valproic and high paracetamol groups (IV) showed similar positive BDNF staining in the hippocampus. p1 value: on comparing sibling of group I and group II, p2 value: on comparing sibling of group I and group III, p3 value: on comparing sibling of group I and group IV, p4 value: on comparing sibling of group II and group III, p5 value: on comparing sibling of group II and group IV, p6 value: on comparing sibling of group III and group IV. *** *p* < 0.001, ns: Not Significant.

**Figure 4:**
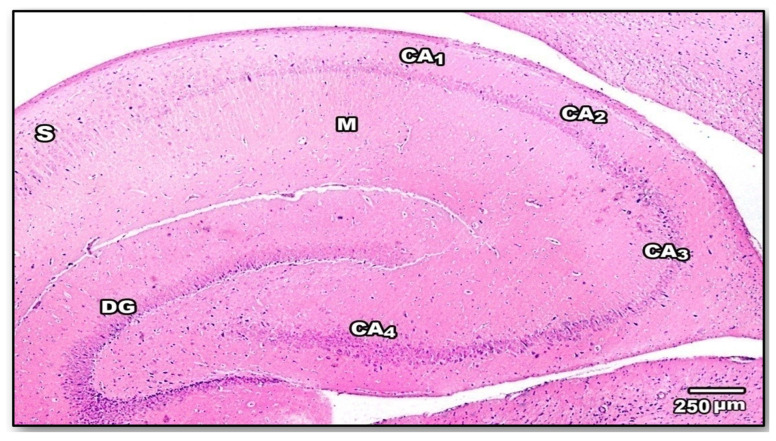
A photomicrograph of a rat hippocampus cut section of group 1 (negative control group) stained with H&E (×40), showing different areas of the hippocampus. The hippocampus proper is formed of the Cornu Ammonis (CA) in the form of CA1, CA2, CA3, and CA4 regions, then continues as the subiculum (S). The dentate gyrus (DG) is seen surrounding CA4 by its upper and lower limbs. Molecular layer (M) was seen inside the concavity of CA and DG.

**Figure 5:**
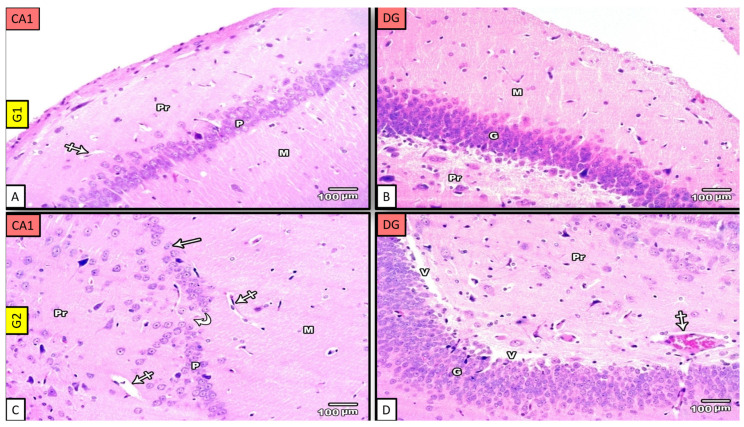

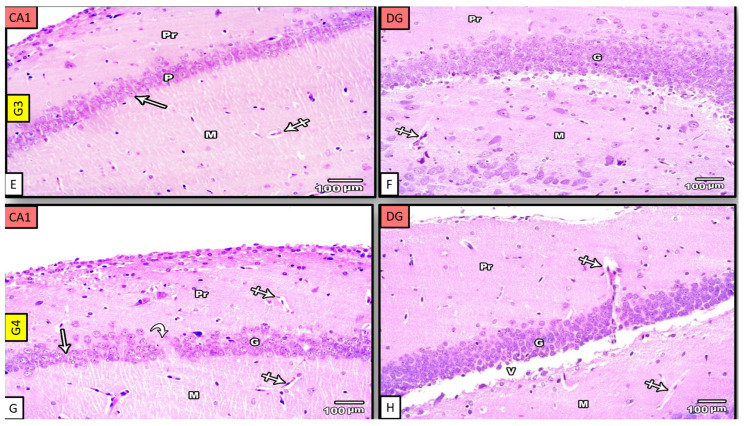
(**A**) A photomicrograph of rat hippocampus cut section from group 1 (negative control group) stained with H&E (×100) showing the three layers of the CA1 region of hippocampus proper: molecular layer (M), pyramidal layer (P), and polymorphic layer (Pr). The pyramidal layer (P) shows layers of small pyramidal cells with vesicular nuclei. Both molecular and polymorphic layers contain blood capillaries (crossed arrow). (**B**) A photomicrograph of rat hippocampus cut section from group 1 (negative control group) stained with H&E (×100) showing dentate gyrus formed of molecular layer (M), granular layer (G), and polymorphic layer (Pr). The granular layer (G) contains rounded granule cells with vesicular nuclei. (**C**) A photomicrograph of rat hippocampus cut section of group 2 (valproic acid-treated group) stained with H&E (×100) showing attenuated irregular pyramidal layer (arrow) with focal cell loss (curved arrow) with normal vasculature (crossed arrows). (**D**) A photomicrograph of a rat hippocampus cut section of group 2 (valproic acid-treated group) stained with H&E (×100) showing dentate gyrus, which appears with a normal thickness of its granular layer and a mildly congested blood vessel (crossed arrows). There is a fissuring tissue artifact which appears as a cleavage line (V). (**E**) A photomicrograph of rat hippocampus cut section of group 3 (therapeutic dose of paracetamol-treated group) stained with H&E (×100) showing the three layers of the CA1 region of hippocampus proper, which appeared normal (arrow) with normal vasculature (crossed arrow). (**F**) A photomicrograph of a rat hippocampus cut section of group 3 (therapeutic dose of paracetamol-treated group) stained with H&E (×100) showing normal dentate gyrus, which appears with normal thickness of its granular layer and normal vasculature (crossed arrow). (**G**) A photomicrograph of rat hippocampus cut section of group 4 (toxic dose of paracetamol-treated group) stained with H&E (×100) showing mild focal attenuation of the pyramidal layer (arrow) with focal cell loss (curved arrow) and normal vasculature (crossed arrows). (**H**) A photomicrograph of a rat hippocampus cut section of group 4 (toxic dose of paracetamol-treated group) stained with H&E (×100) showing dentate gyrus, which appears with normal thickness of its granular layer and normal vasculature (crossed arrows). A fissuring tissue artifact appeared as a cleavage line (V).

**Figure 6:**
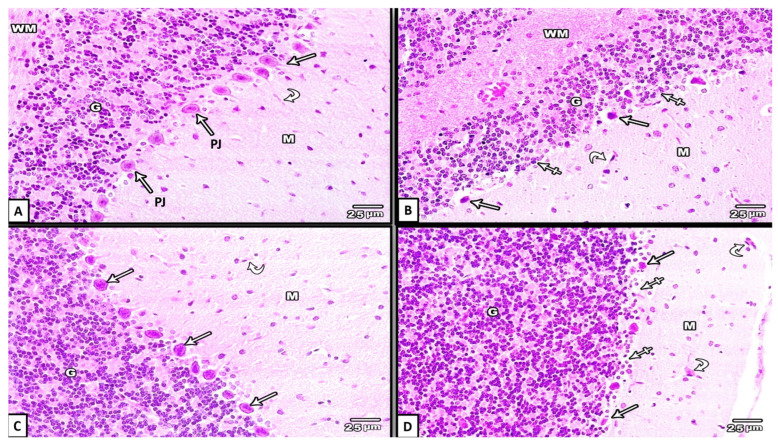
(**A**) A photomicrograph of a rat cerebellum cut section of group 1 (negative control group) stained with H&E (×400), showing different areas of cerebellum. It is formed of 3 layers: outer molecular (M), inner granular cell layer (G), and Purkinje cells (PJ) with flask-shaped cell bodies and centrally located vesicular nuclei (arrows). Blood vessels are seen in the molecular layer (curved arrow). The central medullary region was also seen, made up of white matter (WM). (**B**) A photomicrograph of rat cerebellum cut section of group 2 (valproic acid-treated group) stained with H&E (×400), showing decreased number of the Purkinje cells due to cell loss (crossed arrows) and its shrinkage which became smaller and pyknotic (arrows) with normal vasculature (curved arrow) at the molecular layer but shows mild capillary congestion at the white matter (WM) layer. (**C**) A photomicrograph of rat cerebellum, cut section of group 3 (therapeutic dose of paracetamol-treated group) stained with H&E (×400), showing normal Purkinje cells with flask-shaped bodies and vesicular nuclei (arrows) and normal vasculature (curved arrow). (**D**) A photomicrograph of rat cerebellum cut section of group 4 (toxic dose of paracetamol-treated group) stained with H&E (×400), showing decreased number of the Purkinje cells due to cell loss (crossed arrows) and its shrinkage, which became smaller and pyknotic (arrows) with normal vasculature (curved arrow).

**Figure 7:**
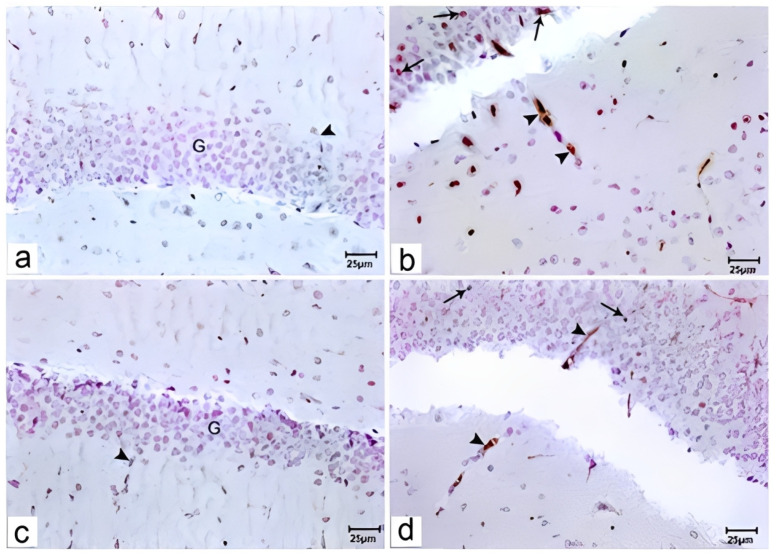
Photomicrograph of hippocampal sections of the offspring of the studied groups stained with immunohistochemical staining for BDNF. (**a**) Immunostained sections of the offspring of group I (negative control group) showing weak focal reaction of BDNF in the cytoplasm of the granular cells (G) or around blood vessels (arrowhead) at the CA3 region ×100. (**b**) Immunostained sections of the offspring of group II (valproic acid-treated group) showing increased BDNF immunoreactivity in the cytoplasm of the granular cells (arrows) and around blood vessels (arrowhead) at the CA3 region ×100. (**c**) Immunostained sections of the offspring of group III (therapeutic dose of paracetamol-treated group) showing weak focal reaction of BDNF in the cytoplasm of the granule cells (G) and around blood vessels (arrowhead) at the CA3 region ×100. (**d**) Immunostained sections of the offspring of group IV (toxic dose of paracetamol-treated group) showing increased BDNF immunoreactivity in the cytoplasm of the granular cells (black arrows) and around blood vessels (black arrowhead) at the CA3 region ×100.

**Figure 8:**
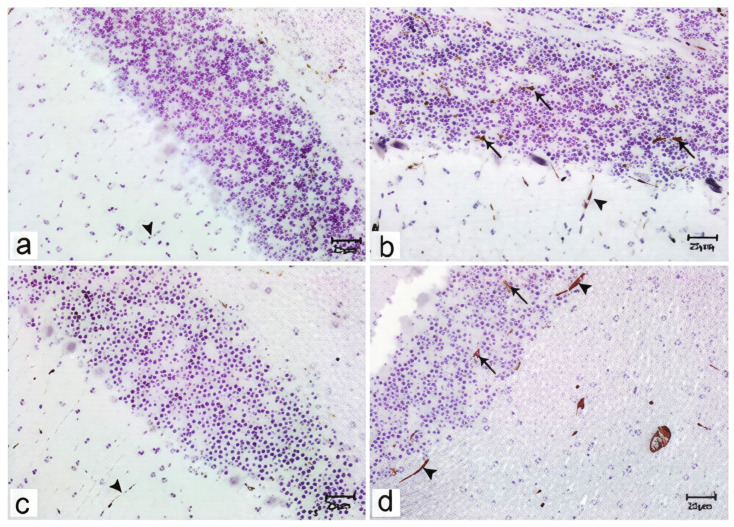
Photomicrograph of cerebellar sections of offspring of the studied groups stained with immunohistochemical staining for BDNF. (**a**) Immune-stained sections of the offspring of group I (negative control group) showing no detectable BDNF expression in the cytoplasm of the Purkinje and granule cells or around blood vessels (arrowhead) ×100. (**b**) Immune-stained sections of offspring of group II (valproic acid-treated group) showing focal increased BDNF expression of granule cells (black arrows) and around blood vessels (black arrowhead), but negative expression at **Purkinje cells ×100.** (**c**) Immune-stained sections of the offspring of group III (therapeutic dose of paracetamol-treated group) showing no detectable BDNF expression in the cytoplasm of the Purkinje and granule cells or around blood vessels (arrowhead) ×100. (**d**) Immune-stained sections of offspring of group IV (toxic dose of paracetamol-treated group) showing no BDNF reactions in the cytoplasm of the Purkinje cells, but granule cells (arrows) and blood vessels showed mild focal positivity (arrowhead) ×100.

**Table 1:** Number of nuclei and average nuclei size at the pyramidal layer of the hippocampal CA1 region in different groups.

Slice (Group)	Count	Average Size in Microns
G1jpg	146	174.4031734
G2jpg	80	170.0244033
G3jpg	156	174.8289667
G4jpg	81	178.5900215

**Table 2:** Average pyramidal layer of hippocampal CA1 region thickness of different groups.

Label	Length in Microns	Average
G1jpg	131.2876	134.096 G1
G1jpg	124.5904	
G1jpg	146.4104	
G2jpg	73.87625	65.0634 G2
G2jpg	62.65977	
G2jpg	58.65447	
G3jpg	118.3033	129.107 G3
G3jpg	151.6361	
G3jpg	117.3829	
G4jpg	66.78226	76.869 G4
G4jpg	94.30646	
G4jpg	69.51958	

## Data Availability

The original contributions presented in this study are included in the article. Further inquiries can be directed to the corresponding author.
